# Ginsenoside Rb1 exerts antiarrhythmic effects by inhibiting I_Na_ and I_CaL_ in rabbit ventricular myocytes

**DOI:** 10.1038/s41598-019-57010-9

**Published:** 2019-12-31

**Authors:** Zhipei Liu, Lv Song, Peipei Zhang, Zhenzhen Cao, Jie Hao, Youjia Tian, Antao Luo, Peihua Zhang, Jihua Ma

**Affiliations:** 1 Cardio-Electrophysiological Research Laboratory, Medical College of Wuhan University of Science and Technology, Wuhan, 430065 China; 2 Hubei Province Key Laboratory of Occupational Hazard Identification and Control, Medical College of Wuhan University of Science and Technology, Wuhan, 430065 China

**Keywords:** Pharmacodynamics, Arrhythmias

## Abstract

Ginsenoside Rb1 exerts its pharmacological action by regulating sodium, potassium and calcium ion channels in the membranes of nerve cells. These ion channels are also present in cardiomyocytes, but no studies have been reported to date regarding the effects of Rb1 on cardiac sodium currents (I_Na_), L-type calcium currents (I_CaL_) and action potentials (APs). Additionally, the antiarrhythmic potential of Rb1 has not been assessed. In this study, we used a whole-cell patch clamp technique to assess the effect of Rb1 on these ion channels. The results showed that Rb1 inhibited I_Na_ and I_CaL_, reduced the action potential amplitude (APA) and maximum upstroke velocity (V_max_), and shortened the action potential duration (APD) in a concentration-dependent manner but had no effect on the inward rectifier potassium current (I_K1_), delayed rectifier potassium current (I_K_) or resting membrane potential (RMP). We also designed a pathological model at the cellular and organ level to verify the role of Rb1. The results showed that Rb1 abolished high calcium-induced delayed afterdepolarizations (DADs), depressed the increase in intracellular calcium ([Ca^2+^]_i_), relieved calcium overload and protected cardiomyocytes. Rb1 can also reduce the occurrence of ventricular premature beats (VPBs) and ventricular tachycardia (VT) in ischemia-reperfusion (I-R) injury.

## Introduction

Panax ginseng Meyer, a traditional herbal medicine, exerts effects of strengthening the body and prolonging life. Modern medical research has shown that it confers protective effects in the nervous system and the cardiovascular system^1–3^ and also exerts antitumoral effects^4^. The main active ingredient of ginseng is ginsenoside, which is divided into two types: protopanaxadiol and protopanaxatriol^5^. Rb1 contains the highest content of protopanaxadiol^6,7^ and has a variety of biological properties, such as antiaging, antiamnestic, anti-inflammatory effects^8,9^. Rb1 has been reported to protect vascular endothelial cells and maintain their normal physiological functions, primarily due to an associated increase in endothelial nitric oxide synthase expression and nitric oxide production^10–12^. Rb1 also inhibits cardiomyocyte apoptosis and death, reducing the area of myocardial infarction caused by ischemia-reperfusion (I-R) injury^13–16^. The above studies indicate that Rb1 has cardiovascular protective function.

Arrhythmia is a severe cardiovascular disease, and ventricular arrhythmias such as ventricular premature beats (VPBs) and ventricular tachycardia (VT) can lead to sudden cardiac death^17,18^. The normal rhythm of the heart is derived from its regular electrical activity, i.e., the transmembrane ion channel currents of cardiomyocytes. The most important currents are I_Na_, I_CaL_ and potassium current. The occurrence of arrhythmias is related to disturbances in cardiac electrical activity. Therefore, many antiarrhythmic drugs act on the above three ion channel currents. Related studies have shown that Rb1 exerts pharmacological effects by regulating sodium ion channels, potassium channels and calcium channels on nerve cell membranes^19–22^. However, whether Rb1 affects the sodium channels, L-type calcium channels and action potentials (APs) of cardiomyocytes has not yet been reported. This study investigated the effects of Rb1 on I_Na_, I_CaL_, potassium current and APs, and explored its potential pharmacological effects of Rb1 against arrhythmia and cardiac cell calcium overload.

## Results

### Effects of Rb1 on I_Na_ in left ventricular myocytes

**Figure 1:**
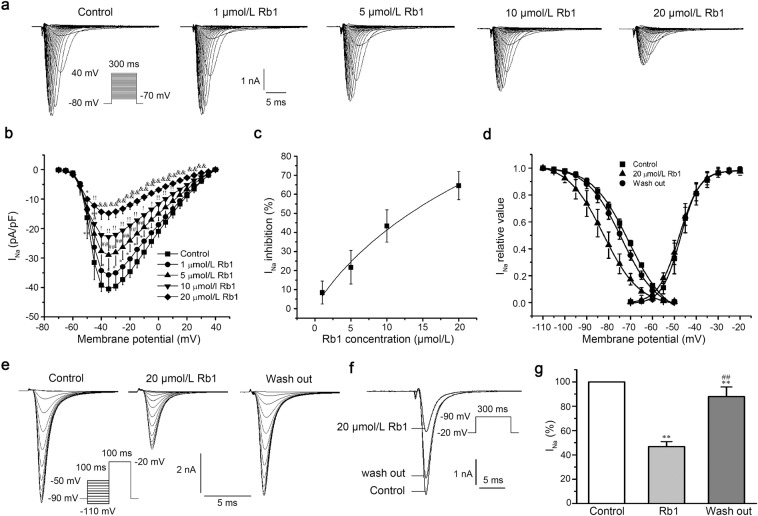
Ginsenoside Rb1 inhibited I_Na_ in a concentration-dependent manner. (**a**) Representative current recordings of I_Na_ in the absence and presence of Rb1. (**b**) The current-voltage relationship of I_Na_ (n = 10, ^*^p < 0.05 vs. control, ^#^p < 0.05 vs. 1 μmol/L Rb1, ^!^p < 0.05 vs. 5 μmol/L Rb1, ^&^p < 0.05 vs. 10 μmol/L Rb1). (**c**) The dose-response relationship of I_Na_. (**d**) The steady-state activation and inactivation curves of I_Na_ in the absence and presence of 20 μmol/L Rb1. (**e**) Representative current recordings of I_Na_ elicited by the pulse in steady-state inactivation curves in the absence and presence of 20 μmol/L Rb1. (**f**) I_Na_ recordings in control conditions, upon addition of 20 μmol/L Rb1, and after Rb1 washout. (**g**) Summary of the data in panel f (n = 11, ^**^p < 0.01 vs. control, ^##^p < 0.01 vs. 20 μmol/L Rb1).

After the addition of Rb1 (1, 5, 10, 20 μmol/L), the I_Na_ decreased in a concentration-dependent manner (n = 10, repeated measures ANOVA, evaluated at −35 mV: Control vs. 1 μmol/L Rb1, p = 4.9E-4; 5 μmol/L Rb1 vs. 1 μmol/L Rb1, p = 2.8E-6; 10 μmol/L Rb1 vs. 5 μmol/L Rb1, p = 1.1E-5; 20 μmol/L Rb1 vs. 10 μmol/L Rb1, p = 5.4E-8; Fig. 1a,b). The half-maximal inhibitory concentration (IC_50_) of I_Na_ was 13 μmol/L (n = 7; Fig. 1c). In the steady-state activation and steady-state inactivation curves of I_Na_ (Fig. 1d), Rb1 (20 μmol/L) shifted the steady-state inactivation curve to the left (more negative membrane potential) and altered the half-inactivation voltage (V_1/2_) from −71 ± 0.55 to −82 ± 0.19 mV (n = 10, repeated measures ANOVA, p = 1.0E-7 < 0.01). When the Rb1 was rinsed off, the half-inactivation voltage returns to −74 ± 0.28 mV (n = 10, repeated measures ANOVA, p = 4.5E-7 < 0.01). However, Rb1 did not affect the steady-state activation curve (n = 16, paired-samples t-test, p = 0.23 > 0.05). Figure 1e shows the inactivation current recordings of I_Na_ under normal conditions with 20 μmol/L Rb1 added and washed out. The results presented in Fig. 1e–g indicated that Rb1 reversibly inhibited I_Na_. After adding 20 μmol/L Rb1, I_Na_ was reduced to 47 ± 4.1% (n = 11, repeated measures ANOVA, p = 0 < 0.01 vs. Control; Fig. 1g). When Rb1 was washed out, I_Na_ was restored to 88 ± 7.9% of the original current (n = 11, repeated measures ANOVA, p = 5.1E-5 < 0.01 vs. Control; p = 0 < 0.01 vs Rb1; Fig. 1g).

### Effects of Rb1 on I_CaL_ in left ventricular myocytes

**Figure 2:**
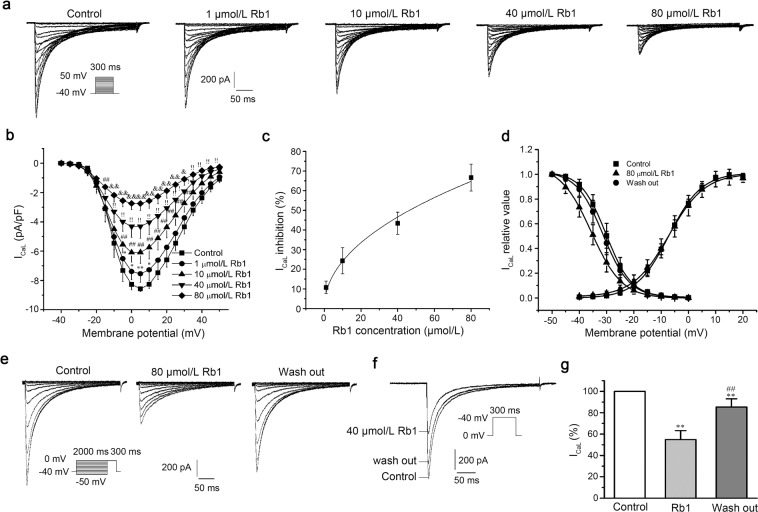
Ginsenoside Rb1 inhibited I_CaL_ in a concentration-dependent manner. (**a**) Representative current recordings of I_CaL_ in the absence and presence of Rb1. (**b**) The current-voltage relationship of I_CaL_ (n = 10, ^*^p < 0.05 vs. control, ^#^p < 0.05 vs^.^ 1 μmol/L Rb1, ^!^p < 0.05 vs. 10 μmol/L Rb1, ^&^p < 0.05 vs. 40 μmol/L Rb1). (**c**) The dose-response relationship of I_CaL_. (**d**) The steady-state activation and inactivation curves of I_CaL_ in the absence and presence of 80 μmol/L Rb1. (**e**) Representative current recordings of I_CaL_ elicited by the pulse in steady-state inactivation curves in the absence and presence of 80 μmol/L Rb1. (**f**) I_CaL_ recordings in control conditions, upon addition of 40 μmol/L Rb1, and after Rb1 washout. (**g**) Data summary of panel f (n = 8, ^**^p < 0.01 vs. control, ^##^p < 0.01 vs. 40 μmol/L Rb1).

After the addition of Rb1 (1, 10, 40, 80 μmol/L), I_CaL_ decreased in a concentration-dependent manner (n = 10, repeated measures ANOVA, evaluated at 5 mV: Control vs. 1 μmol/L Rb1, p = 2.1E-3; 10 μmol/L Rb1 vs. 1 μmol/L Rb1, p = 1.2E-5; 40 μmol/L Rb1 vs. 10 μmol/L Rb1, p = 2.5E-7; 80 μmol/L Rb1 vs. 40 μmol/L Rb1, p = 3.4E-6; Fig. 2a,b). The IC_50_ of I_CaL_ was 42 μmol/L (n = 6; Fig. 2c). Rb1 (80 μmol/L) shifted the steady-state inactivation curve of I_CaL_ to the left (more negative membrane potential) and altered the V_1/2_ from −30 ± 0.10 to −35 ± 0.12 mV (n = 10, repeated measures ANOVA, p = 1.4E-6 < 0.01); When the Rb1 was rinsed off, the half-inactivation voltage returns to −31 ± 0.10 mV (n = 10, repeated measures ANOVA, p = 3.4E-6 < 0.01). However, Rb1 did not affect the steady-state activation curve of I_CaL_ (n = 12, paired-samples t-test, p = 0.93 > 0.05; Fig. 2d). Figure 2e shows the inactivation current recordings of I_CaL_ under normal conditions with 80 μmol/L Rb1 added and washed out. The results presented in Fig. 2e–g indicate that Rb1 reversibly inhibited I_CaL_. After adding 40 μmol/L Rb1, I_CaL_ was reduced to 55 ± 8.4% (n = 8, repeated measures ANOVA, p = 0 < 0.01 vs. Control; Fig. 2g). When Rb1 was washed out, I_CaL_ returned to 85 ± 7.7% of the original current (n = 8, repeated measures ANOVA, p = 5.2E-4 < 0.01 vs. Control; p = 3.0E-7 < 0.01 vs. Rb1; Fig. 2g).

### Effects of Rb1 on I_K1_ and I_K_ in left ventricular myocytes

**Figure 3:**
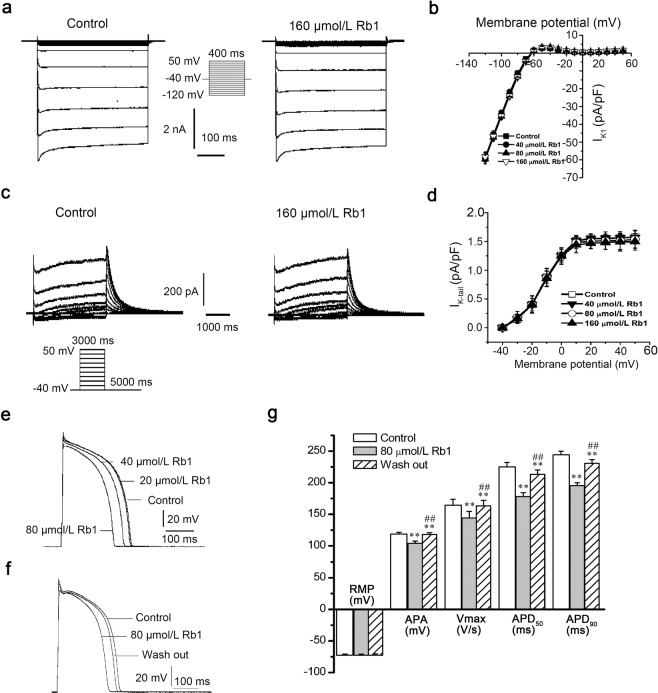
Effects of ginsenoside Rb1 on I_K1_, I_K_ and AP. (**a**) Representative current recordings of I_K1_ in the absence and presence of 160 μmol/L Rb1. (**b**) The current-voltage relationship of I_K1_ under different conditions (control and 40, 80, and 160 μmol/L Rb1). (**c**) Representative current recordings of I_K_ in the absence and presence of 160 μmol/L Rb1. (**d**) The current-voltage relationship of I_K_ under different conditions (control and 40, 80, and 160 μmol/L Rb1). (**e**,**f**) Representative AP recordings in the absence and presence of Rb1 (20, 40, and 80 μmol/L). (**g**) Data summary of panel f (n = 7, ^**^p < 0.01 vs. control, ^##^p < 0.01 vs^.^ 40 μmol/L Rb1).

Rb1 (160 μmol/L) had no effect on I_K1_ and I_K_ (Fig. 3a,c). The current-voltage curve under normal conditions and after the addition of various concentrations of Rb1 (40, 80, 160 μmol/L) completely coincided, indicating that Rb1 had no effect on I_k1_ (n = 10, repeated measures ANOVA, evaluated at −120 mV: Control vs. 40 μmol/L Rb1, p = 0.39; Control vs. 80 μmol/L Rb1, p = 0.11; Control vs. 160 μmol/L Rb1, p = 0.99, Fig. 3b) or I_K_ (n = 8, repeated measures ANOVA, evaluated at 50 mV: Control vs. 40 μmol/L Rb1, p = 0.99; Control vs. 80 μmol/L Rb1, p = 0.28; Control vs. 160 μmol/L Rb1, p = 0.67; Fig. 3d).

### Effects of Rb1 on AP and DADs in left ventricular myocytes

**Figure 4:**
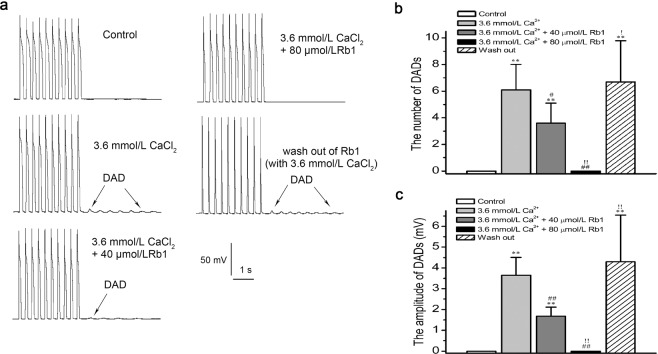
Effects of ginsenoside Rb1 on high calcium-induced DADs. (**a**) Ginsenoside Rb1 abolished high calcium-induced DADs. (**b**,**c**) Statistical analysis of the number and magnitude of DADs (n = 10, ^**^p < 0.05 vs. control, ^#^p < 0.05 and ^##^p < 0.01 vs. 3.6 mmol/L Ca^2+^, ^!^p < 0.05 and ^!!^p < 0.01 vs. 3.6 mmol/L Ca^2+^  + 40 μmol/L Rb1).

Rb1 (20, 40, 80 μmol/L) reduced the action potential amplitude (APA) (n = 10, repeated measures ANOVA: Control vs. 40 μmol/L Rb1, p = 2.3E-2 < 0.05; 80 μmol/L Rb1 vs. 40 μmol/L Rb1, p = 2.2E-2 < 0.05) and maximum upstroke velocity (V_max_) (n = 10, repeated measures ANOVA: Control vs. 40 μmol/L Rb1, p = 4.5E-3 < 0.01; 40 μmol/L Rb1 vs. 20 μmol/L Rb1, p = 4.2E-3 < 0.01; 80 μmol/L Rb1vs. 40 μmol/L Rb1, p = 3.5E-3 < 0.01) and shortened the action potential duration at 50% of repolarization (APD_50_) (n = 10, repeated measures ANOVA: 40 μmol/L Rb1 vs. 20 μmol/L Rb1, p = 1.3E-2 < 0.05; 80 μmol/L Rb1 vs. 40 μmol/L Rb1, p = 3.8E-2 < 0.05) and APD at 90% of repolarization (APD_90_) (n = 10, repeated measures ANOVA: 40 μmol/L Rb1 vs. 20 μmol/L Rb1, p = 7.6E-4 < 0.01; 80 μmol/L Rb1 vs. 40 μmol/L Rb1 p = 2.8E-7 < 0.01) but did not affect the resting membrane potential (RMP) (n = 10, repeated measures ANOVA: 20 μmol/L Rb1 vs. Control, p = 0.99 > 0.05; 40 μmol/L Rb1 vs. Control, p = 0.98 > 0.05; 80 μmol/L Rb1 vs. Control, p = 0.99 > 0.05; Table 1 and Fig. 3e). We also tested whether the effect of Rb1 on APs is reversible. In the presence of 80 μmol/L Rb1, the APA and Vmax values decreased and the APD_50_ and APD_90_ values shortened. In addition, these parameters were tested after Rb1 was washed out of the cells (n = 7, repeated measures ANOVA, 80 μmol/L Rb1 vs. wash out: APA, p = 5.6E-8 < 0.01; Vmax, p = 0 < 0.01; APD_50_, p = 0 < 0.01; APD_90_, p = 0 < 0.01; Fig. 3f,g). As shown in Fig. 4a, under a basic cycle length of 300 ms and a string stimulation interval of 0.125 Hz, 10 consecutive APs were recorded. When the recording was stable, we used a high calcium (the Ca^2+^ concentration of the extracellular fluid was 3.6 mmol/L) solution for perfusion. Then, delayed afterdepolarizations (DADs) were observed in all 10 cells assayed (n = 10, paired-samples t-test, Control vs. 3.6 mmol/L Ca^2+^: number of DADs, p = 3.3E-6 < 0.01; amplitude, p = 2.9E-7 < 0.01; Fig. 4). The DADs were depressed after the addition of 40 μmol/L Rb1 (n = 10, paired-samples t-test, 40 μmol/L Rb1 vs. 3.6 mmol/L Ca^2+^: number of DADs, p = 1.9E-2 < 0.05; amplitude, p = 7.9E-6 < 0.01) and disappeared after adding 80 μmol/L Rb1 (n = 10, paired-samples t-test, 80 μmol/L Rb1 vs. 40 μmol/L Rb1: number of DADs, p = 3.5E-5 < 0.01; amplitude, p = 7.5E-7 < 0.01). When the Rb1 was washed away with high calcium perfusate, the DADs reappeared (n = 10, paired-samples t-test, wash out vs. 3.6 mmol/L Ca^2+^: number of DADs, p = 0.55 > 0.05; amplitude, p = 0.32 > 0.05).

**Table 1:** Effects of Rb1 (20, 40, and 80 μmol/L) on action potentials.

	Control	Rb1 (μmol/L)		
		20	40	80
RMP (mV)	76.9 ± 2.4	77.0 ± 2.1	76.8 ± 2.3	76.9 ± 2.8
APA (mV)	132.8 ± 4.6	129.7 ± 6.8	126.0 ± 6.2^*^	118.7 ± 4.9^**##†^
V_max_ (V/s)	197.0 ± 8.6	194.7 ± 7.2	189.0 ± 9.0^**##^	178.1 ± 10.4^##††^
APD_50_	219.9 ± 11.5	216.0 ± 14.5	195.2 ± 18.6^**#^	176.4 ± 15.9^**##†^
APD_90_	242.6 ± 15.0	237.6 ± 19.4	215.6 ± 19.4^**##^	189.4 ± 20.8^##††^

Note: RMP = resting membrane potential; APA = action potential amplitude; V_max_ = maximum upstroke velocity; APD_50_ = action potential duration at 50% repolarization; APD_90_ = action potential duration at 90% repolarization. The data are expressed as the mean ± SD (n = 10, stimulation frequency = 1 Hz). ^**^P < 0.01 vs. Control, ^*^P < 0.05 vs. Control, ^##^P < 0.01 vs. 20 μmol/L, ^#^P < 0.05 vs. 20 μmol/L, ^†^P < 0.05 vs. 40 μmol/L, ^††^P < 0.01 vs. 40 μmol/L.

### Effects of Rb1 on [Ca^2+^]_i_ under hypoxia/reoxygenation (H-R) conditions

**Figure 5:**
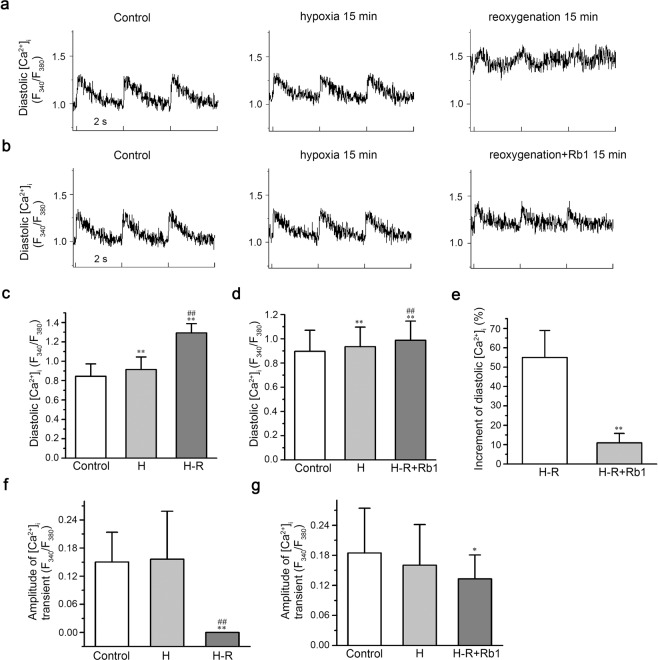
Effects of ginsenoside Rb1 on [Ca^2+^]_i_ under H-R conditions. (**a**,**b**) Representative recordings of [Ca^2+^]_i_ transients under H-R in the H-R group and the Rb1 group. (**c**,**f**) Diastolic [Ca^2+^]_i_ and amplitude of [Ca^2+^]_i_ transients in the H-R group during different time periods (n = 7, ^**^P < 0.01 vs. control, ^##^P < 0.01 vs. H). (**d**,**g**) Diastolic [Ca^2+^]_i_ and amplitude of [Ca^2+^]_i_ transients in the Rb1 group during different time periods (n = 8, ^**^P < 0.01 vs. control, ^*^P < 0.05 vs. control, ^##^P < 0.01 vs^.^ H). (**e**) Changes in diastolic [Ca^2+^]_i_ during H-R in the two groups.

**Figure 6:**
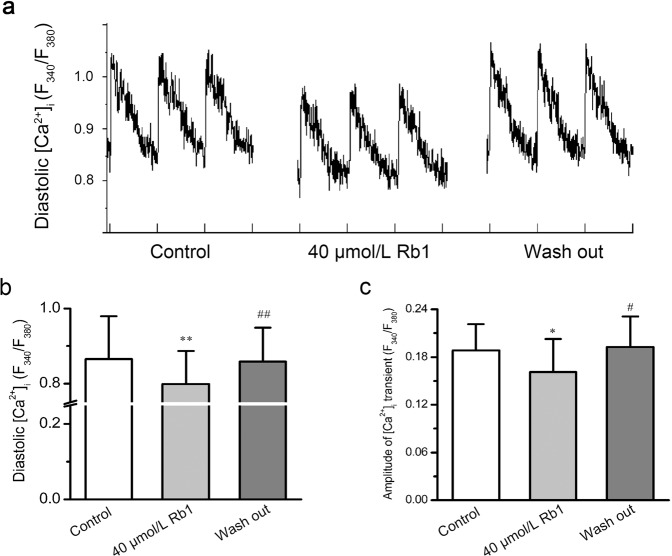
Effects of ginsenoside Rb1 on [Ca^2+^]_i_ under normal conditions. (**a**) Representative recordings of [Ca^2+^]_i_ transients in the absence and presence of Rb1. (**b**,**c**) Diastolic [Ca^2+^]_i_ and amplitude of [Ca^2+^]_i_ transients in the absence and presence of Rb1 (n = 10, ^*^P < 0.05 and ^**^P < 0.01 vs^.^ control, ^#^p < 0.05 and ^##^P < 0.01 vs. 40 μmol/L Rb1).

The effects of Rb1 on [Ca^2+^]_i_ under H-R conditions was assessed in two groups, the H-R group and the Rb1 group. Both groups underwent normal perfusion for 5 min, hypoxia for 15 min and reperfusion for 15 min. The H-R group was reperfused with normal perfusate, while the Rb1 group was reperfused with perfusate containing 40 μmol/L Rb1. After 15 min of hypoxia in both groups, the diastolic [Ca^2+^]_i_ increased slowly (paired-samples t-test, H-R group, n = 7, p = 2.0E-3 < 0.01; Rb1 group, n = 8, p = 7.4E-3 < 0.01), but the Δ[Ca^2+^]_i_ did not significantly change (paired-samples t-test, H-R group, n = 7, p = 0.76 > 0.05; Rb1 group, n = 8, p = 0.25 > 0.05). After 15 min of reoxygenation, the diastolic [Ca^2+^]_i_ of the H-R group increased rapidly (n = 7, paired-samples t-test: p = 4.8E-4 < 0.01, H-R vs. Control; p = 8.0E-4 < 0.01, H-R vs. H; Fig. 5a,c) and the Δ[Ca^2+^]_i_ decreased significantly (n = 7, paired-samples t-test: p = 1.4E-4 < 0.01, H-R vs. Control; p = 1.1E-3 < 0.01, H-R vs. H; Fig. 5a,f). Compared with the H-R group, the diastolic [Ca^2+^]_i_ of the Rb1 group increased slowly (n = 8, paired-samples t-test: p = 3.8E-5 < 0.01, H-R + Rb1 vs. Control; p = 8.0E-5 < 0.01 H-R + Rb1 vs. H; Fig. 5b,d,), and the Δ[Ca^2+^]_i_ did not significantly decrease (n = 8, paired-samples t-test, p = 1.6E-2 < 0.05, H-R + Rb1 vs. Control; p = 0.20 > 0.05, H-R + Rb1 vs. H; Fig. 5b,g). After 15 minutes of reoxygenation, the diastolic [Ca^2+^]_i_ of the H-R group increased by 55 ± 14% compared with that observed under normal perfusion, while the diastolic [Ca^2+^]_i_ of the Rb1 group increased by 11 ± 4.9% compared with that observed under normal perfusion. In contrast, the diastolic [Ca^2+^]_i_ of the Rb1 group increased slowly compared with that observed the H-R group (two-samples t-test, p = 4.6E-4 < 0.01, H-R group vs. Rb1 group; Fig. 5e). These results indicated that Rb1 can inhibit calcium overload induced by H-R. In the I-R group, all cells showed spontaneous contraction and rhythm disorder after reoxygenation (7/7), while the cells in the Rb1 group showed normal contraction after reoxygenation (0/8). These results indicated that Rb1 has myocardial protective function. Figure 5a,b shows images of calcium transients for the two experimental groups under normoxic conditions, after 15 min of hypoxia and after 15 min of reoxygenation. We also tested the effects of Rb1 on the diastolic [Ca^2+^]_i_ and Δ[Ca^2+^]_i_, observing that 40 μmol/L Rb1 reduced the diastolic [Ca^2+^]_i_ and decreased Δ[Ca^2+^]_i_ (n = 6, repeated measures ANOVA, 40 μmol/L Rb1 vs. Control; [Ca^2+^]_i_, p = 4.7E-4 < 0.01; Δ[Ca^2+^]_i_, p = 1.1E-2 < 0.05; Fig. 6). This effect was reversible (n = 6, repeated measures ANOVA, Wash out vs. Control: [Ca^2+^]_i_, p = 0.84 > 0.05; Δ[Ca^2+^]_i_, p = 0.21 > 0.05).

### Effects of Rb1 on I-R -induced ventricular arrhythmia

**Figure 7:**
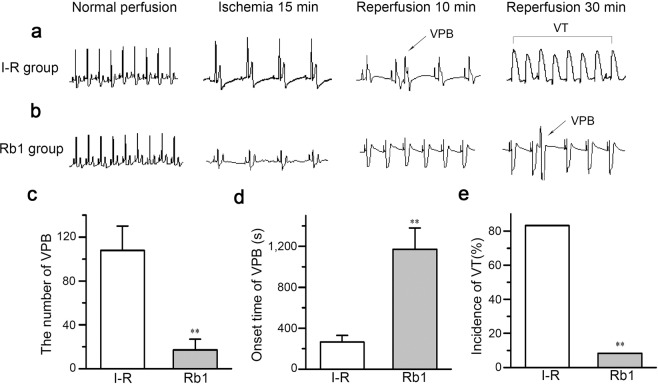
Effects of ginsenoside Rb1 on I-R-induced ventricular arrhythmia. (**a**) Representative ECG recordings from the I-R group at different time periods. (**b**) Representative ECG recordings from the Rb1 group at different times periods. (**c**) The number of VPBs that occurred during the reperfusion periods (n = 12, ^**^p < 0.01 vs. I-R group). (**d**) The onset time of VPBs in the reperfusion periods (n = 12, ^**^p < 0.01 vs^.^ I-R group). (**e**) The incidence of VT occur in the I-R group and the Rb1 group (n = 12, ^**^p < 0.01 vs. I-R group).

Two groups were assessed for the effects of Rb1 on I-R -induced ventricular arrhythmia, which was produced subjecting the groups to Langendorff heart perfusion for 10 min, ischemia for 30 min and reperfusion for 60 min. The I-R group was reperfused with fresh Tyrode’s solution, and the Rb1 group was reperfused with Tyrode’s solution containing 40 μmol/L Rb1. In the I-R group, the number of VPBs during reperfusion was 108 ± 22, and the onset time of VPBs was 264 ± 66 s. In the Rb1 group, the number of VPBs decreased to 17 ± 9.6 and the onset time was extended to 1172 ± 208 s (two-sample t-test, n = 12, VPB number: p = 1.5E-9 < 0.01, I-R group vs. Rb1 group; VPB onset time: p = 6.9E-8 < 0.01, I-R group vs. Rb1 group; Fig. 7c,d). VT occurred in 10 of 12 hearts in the I-R group (83%), but in only 1 heart (1/12, 8%) in the Rb1 group (Fisher’s exact probability, n = 12, p = 6.4E-4 < 0.01, I-R group vs. Rb1 group; Fig. 7e). Figure 7a,b shows ECG recordings from the two groups over four specific time periods.

## Discussion

In this study, we observed that Rb1 can inhibit I_Na_ and I_CaL_ in a concentration-dependent manner and that this effect is reversible. Rb1 had no effect on the activation of these two currents, but could shift the inactivation curve to the left (negative potential) and accelerate their deactivation. The APA, V_max_, APD_50_ and APD_90_ values decreased in the presence of Rb1, but the RMP, I_K1_ and I_K_ were not affected by Rb1. In addition, Rb1 could alleviate the increase in diastolic [Ca^2+^]_i_ induced by H-R and inhibit the spontaneous contraction and death of cardiomyocytes due to calcium overload. Rb1 could also reduce the occurrence of VPBs and VT caused by I-R injury.

Voltage-gated sodium channels play an indispensable role in the excitability and conduction of ventricular myocytes. Their opening is rapid and constitutes the rising phase of the AP of ventricular myocytes. Inhibition of I_Na_ can increase the threshold for triggering an AP and reduce the occurrence of transient depolarization, reducing the risk of arrhythmia^23^. Rb1 was shown to reduce I_Na_ (Fig. 1a) and shift the steady-state inactivation curve to the left (Fig. 1d), which has a similar effect as class I antiarrhythmic drugs. Class I antiarrhythmic drugs can inhibit arrhythmia by increasing the threshold of action potential, reducing abnormal automaticity, slowing down abnormal conductivity and prolonging the effective refractory period. Due to its inhibition of sodium current, the excitatory conduction slows down and leads to arrhythmia. Therefore, Rb1 may also have the same arrhythmogenic effect as a class I antiarrhythmic drug.

The I_CaL_ is an inward current that constitutes the plateau of ventricular myocytes. The maintenance of the plateau period depends on the balance between the outward current and the inward current. When the I_CaL_ increases, the repolarization reserve decreases and the APD extends. The extended plateau provides conditions for the reactivation of the calcium channels, which produces early afterdepolarizations (EADs) that induces arrhythmia^24^. Rb1 was observed to inhibit I_CaL_ (Fig. 2a) and accelerate the inactivation of calcium channels (Fig. 2d), reducing the flow of calcium ions into cells. Therefore, Rb1 reduces [Ca^2+^]_i_ overload caused by H-R and inhibits arrhythmias, as [Ca^2+^]_i_ overload is an important factor in inducing arrhythmia^25,26^.

The AP is a comprehensive representation of the ion current of cardiomyocytes, and changes in ion currents affect the shape of APs. Rb1 reduced the APA and V_max_ values, which was the result of I_Na_ inhibition. When V_max_ is reduced, cell excitability and conductivity are reduced, which can decrease the occurrence of ectopic excitability. The plateau of the AP is primary formed by the inward I_CaL_ and the outward I_K._ Interestingly, Rb1 suppressed I_CaL_ but had no effect on I_K_. In general, the inward current was reduced, and the APD was shortened, which is beneficial for reducing the occurrence of EADs and inhibiting long QT syndrome-induced arrhythmias^27,28^. I_K1_ is involved in the formation of the RMP, and because Rb1 had no effect on I_K1_ (Fig. 3a), it did not alter the RMP (Table 1). Rb1 has been reported to increase the slow component of delayed rectifier potassium current (I_Ks_) in guinea pig ventricular myocytes^29^, which contradicts the results of the present study, and this contradiction may be caused by differences in the studied species.

Ca^2+^ is an extremely important second messenger in the cell, participating in many physiological activities, such as contraction, secretion, gene expression and so on^30^. Under normal conditions, [Ca^2+^]_i_ influx and outflow are equal. When the inflow increases or the outflow decreases, [Ca^2+^]_i_ increases, causing calcium overload. Calcium overload can overactivate a variety of enzymatic reactions, affecting the normal physiological functions of cells^31^. Calcium overload also causes the sarcoplasmic reticulum calcium content to increase. Beyond a specific certain limit, the sarcoplasmic reticulum spontaneously releases Ca^2+^, causing DADs and posterior contraction and eventually leading to arrhythmia^32^. In this study, we showed that Rb1 significantly inhibits DADs induced by high calcium concentrations in ventricular myocytes and exerts anti-arrythmia activity in cardiac cells. The entry and outflow of Ca^2+^ is essential for maintaining the normal physiological activities of cells. With respect to hypoxia, ischemia and heart failure, [Ca^2+^]_i_ increases and calcium overload occurs^33^. After 15 min of hypoxia and 15 min of reoxygenation in the H-R group, diastolic [Ca^2+^]_i_ increased significantly and a contraction rhythm disorder appeared, which was the result of calcium overload. In the Rb1 group, diastolic [Ca^2+^]_i_ was also increased after hypoxia and reoxygenation with additional Rb1. However, the percentage of increase was lower than that observed in the H-R group, and a systolic rhythm disorder was not observed. This result indicates that Rb1 can inhibit the increase in diastolic [Ca^2+^]_i_ caused by H-R, thereby inhibiting calcium overload, maintaining normal contraction rhythm of cardiomyocytes and protecting cardiomyocytes from H-R. This result may be related to the inhibition of I_CaL_.

All of the above experiments only explored the role of Rb1 at the cellular level and used unilateral indicators as a reference. To assess whether Rb1 has an antiarrhythmic effect, we performed an organ-level experiment. I-R injury can lead to disturbances in various currents in cardiomyocytes, thereby inducing ventricular arrhythmias^18^. In these experiments, we observed that the incidence of VPBs and VT was significantly reduced and that the onset time was delayed when hearts were reperfused with Rb1-containing perfusate (Fig. 7), indicating that Rb1 indeed confers resistance to arrhythmias.

Ginsenoside Rb1 can simultaneously inhibit I_CaL_ and I_Na_, which acts to lower the APA, reduce V_max_, and shorten the APD of APs. Due to its effect on I_CaL_, Rb1 inhibits high calcium-induced DADs, cellular calcium overload induced by H-R. Due to its effect on both I_CaL_ and I_Na_, Rb1 reduces ventricular arrhythmias induced by I-R injury, which may be the reason for its antiarrhythmic effect.

## Materials and Methods

### Preparation of ventricular myocytes

The animals used in this experiment are in line with the “Guidelines for the Care and Use of Laboratory Animals” formulated by Hubei Province, China, and approved by the Institutional Animal Care and Use Committee of Wuhan University of Science and Technology.

New Zealand white rabbits weighing 1.5–2 kg were screened as the experimental subjects (male and female had no effect on the experimental results). The rabbits were heparinized (2000 U) and anesthetized with xylazine (7.5 mg/kg i.m.) and ketamine (30 mg/kg, i.v.). After the heart was removed, the aorta was cannulated. Next, we fixed the heart on a Langendoff apparatus and retrogradely perfused it with Ca^2+^-free Tyrode’s solution for 5 min to discharge the heart congestion. The solution was then changed to enzyme-containing Ca^2+^-free Tyrode’s solution (collagenase 1 g/L, bovine serum albumin, BSA 1 g/L). After 40 min of perfusion, we used Kraft-Brühe (KB) solution to irrigate the heart to discharge residual enzymes. All solutions were preoxygenated (95% O_2_ and 5% CO_2_) and maintained at 37 °C. After removing the heart, the left ventricle was cut and placed in a small beaker containing KB solution. The cells were filtered through nylon mesh and stored in KB solution at 4 °C for later use.

### Drugs and solutions

Collagenase type I was purchased from Gibco (GIBCO TM, Invitrogen, Paisley, UK). HEPES and BSA were obtained from Roche (Basel, Switzerland). Ginsenoside Rb1, Fura-2/AM and other drugs were purchased from Sigma Aldrich (Saint Louis, MO, USA). Rb1 was dissolved in methanol, and tests were performed that excluded the role of solvent in the observed effects.

The Ca^2+^-free Tyrode’s solution contained (in mmol/L) 135 NaCl, 5.4 KCl, 1 MgCl_2_, 0.33 NaH_2_PO_4_, 10 glucose and 10 HEPES (pH 7.4).

The KB solution contained (in mmol/L) 70 KOH, 40 KCl, 20 KH_2_PO_4_, 3 MgSO_4_, 50 glutamic acid, 20 taurine, 10 glucose, 0.5 EGTA and 10 HEPES (pH 7.4).

For I_Na_ recording, the bath solution contained (in mmol/L) 105 CsCl, 30 NaCl, 1 MgCl_2_, 1 CaCl_2_, 0.05 CdCl_2_, 5 glucose and 5 HEPES (pH 7.4), and 0.01 mmol/L nifedipine was added to the solution to block L-type Ca^2+^channels. The pipette solution contained (in mmol/L) 120 CsCl, 5 Na_2_ATP, 5 MgCl_2_, 1 CaCl_2_, 10 TEA-Cl, 10 EGTA and 10 HEPES (pH 7.3).

For I_CaL_ recording, the bath solution contained (in mmol/L) 135 NaCl, 5.4 CsCl, 1.8 CaCl_2_, 1 MgCl_2_, 0.3 BaCl_2_, 0.33 NaH_2_PO_4_, 10 glucose and 10 HEPES (pH 7.4). The pipette solution contained (in mmol/L) 120 CsCl, 5 Na_2_ATP, 5 MgCl_2_, 1 CaCl_2_, 10 TEA-Cl, 10 EGTA and 10 HEPES (pH 7.3).

For I_K1_ recording, the bath solution contained (in mmol/L) 137 NaCl, 5.4 KCl, 1.8 CaCl_2_, 1 MgCl_2_, 0.33 NaH_2_PO_4_, 0.3 CdCl_2_, 10 glucose and 10 HEPES (pH 7.35). The pipette solution contained (in mmol/L) 140 KCl, 1 MgCl_2_, 5 K_2_ATP, 10 EGTA and 5 HEPES (pH 7.3).

For I_K_ recording, the bath solution contained (in mmol/L) 135 NaCl, 5.4 KCl, 1 MgCl_2_, 1 CaCl_2_, 0.33 NaH_2_PO_4_, 0.2 CdCl_2_, 5 glucose and 5 HEPES (pH 7.4). The pipette solution contained (in mmol/L) 140 KCl, 1 MgCl_2_, 2 Na_2_ATP, 10 EGTA and 5 HEPES (pH 7.25).

For AP recording, the bath solution contained (in mmol/L) 145 NaCl, 5.4 KCl, 1.2 MgCl_2_, 1.8 CaCl_2_, 10 glucose and 5 HEPES (pH 7.4). The pipette solution contained (in mmol/L) 110 K-aspartate, 30 KCl, 5 NaCl, 5 creatine phosphate, 5 MgATP, 0.05 cAMP, 0.1 EGTA and 10 HEPES (pH 7.3).

For [Ca^2+^]_i_ transient recording, the bath solution contained (in mmol/L) 131 NaCl, 4 KCl, 1.8 CaCl_2_, 1 MgCl_2_, 10 glucose and 10 HEPES (pH 7.4). The solution used during hypoxia was without glucose and bubbled with 100% N_2_.

### Ion current and AP recording

Temperatures were maintained at 22–25 °C in all experiments. The glass electrodes were pulled with a two-stage patch pipette puller (PP-830, Narishige Group, Tokyo, Japan) and thermally polished. The electrode resistance was 1.5–2 MΩ when filled with pipette solution. Currents and APs were recorded by an EPC-10 patch clamp amplifier (HEKA Electronic, Lambrecht, Pfalz, Germany), filtered at 2 kHz and sampled at 10 kHz.

I_Na_ was elicited by a 0.2 Hz and 300 ms depolarizing pulse from a holding potential of −90 mV to −20 mV. To record the current-voltage relationship of I_Na_, the depolarizing pulse was changed from −70 mV to +40 mV in 5-mV increments at a frequency of 0.5 Hz. For the steady-state I_Na_ inactivation curve, currents were evoked by a 100 ms conditioning prepulse from −100 mV to −50 mV in 5 mV increments at a holding potential of −90 mV followed by a 100 ms depolarizing test pulse to −30 mV. The frequency was 0.5 Hz.

I_CaL_ was elicited by a 0.2 Hz and 300 ms depolarizing pulse from a holding potential of −40 mV to 0 mV. To record the current-voltage relationship of I_CaL_, the depolarizing pulse was changed from −40 mV to +50 mV in 5 mV increments at a frequency of 0.5 Hz. For the steady-state I_CaL_ inactivation curve, currents were evoked by a 2000 ms conditioning prepulse from −50 mV to 0 mV in 5-mV increments at a holding potential of −40 mV followed by a 300 ms depolarizing test pulse to 0 mV. The frequency was 0.5 Hz.

I_K1_ was elicited by a 1 Hz and 400 ms depolarizing pulse from −120 mV to +50 mV in 10 mV increments at a holding potential of −40 mV.

I_K_ was elicited by a 0.1 Hz and 3000 ms depolarizing pulse from −40 mV to +50 mV in 10 mV increments followed by a 5000 ms repolarization pulse to −40 mV at a holding potential of −40 mV.

APs were elicited by 5 ms duration and current pulses of 1.5 times the diastolic threshold at a frequency of 1 Hz using the patch clamp technique in current clamp mode. The RMP, APA, V_max_, APD_50_ and APD_90_ parameters were assessed.

### Determination of ventricular myocyte [Ca^2+^]_i_ transients

The cell suspension was incubated with 1 μmol/L Fura-2/AM for 30 min in the dark at 25 °C. The cells that had been loaded with Fura-2 were placed in a cell pool on an inverted microscope (Olympus IX-70) and stimulated with a platinum electrode (0.5 Hz, 37 °C). The experiments were performed on cardiac myocytes with the appropriate morphological appearance (rod shape, sharp edges, clear stripes), sarcomere length greater than 1.70 μm and no spontaneous contractions. Cardiac myocyte [Ca^2+^]_i_ transients were measured using a dual-excitation fluorescence photomultiplier system (IonOptix, Milton, MA, USA). Xenon lamps provide excitation light with alternating excitation wavelengths of 340 nm or 380 nm at 250 Hz. The photomultiplier tube collects the emitted fluorescent signals and continuously measures the ratio of the Fura-2 fluorescent signals (F340/F380) after subtraction of the background fluorescence. The drug was applied after stabilization and the magnitudes of diastolic [Ca^2+^]_i_ (F_340_/F_380_) and the amplitude of the [Ca^2+^]_i_ transient (Δ[Ca^2+^]_i_; F_340_/F_380_) were measured.

### Electrocardiogram (ECG) recording

Hearts were obtained as described in the preparation of ventricular myocytes. We fixed the heart on a Langendoff apparatus and retrogradely perfused it with Ca^2+^-free Tyrode’s solution. Three silver electrodes were placed on the heart to elicit the ECG. The ECG was recorded and measured using a multichannel physiological signal acquisition and processing system (RM6240C, Chengdu Instrument Factory, Sichuan, China). After ECG stabilization, we perfused the heart with normal Tyrode’s solution for 10 min and then stopped perfusion. We reperfused the heart 30 min later with fresh Tyrode’s solution or Tyrode’s solution containing 40 μmol/L Rb1 for 60 min. We counted the number, VPB onset time and occurrence of VT during 60 min of reperfusion. Five consecutive VPBs are considered VT.

### Data analysis

Data analysis was performed using FitMaster (v2x32, HEKA). Statistical analyses were performed using Origin 8.0 (OriginLab, Northampton, MA). The data are presented as the mean ± SD. Two-tailed t-tests were used to compare the two groups, and a repeated measures ANOVA was used to compare multiple groups. P < 0.05 was considered statistically significant. Both the steady-state inactivation curve and the steady-state activation curve were fitted to the Boltzmann equation, Y = 1/[1 + exp^(Vm-V1/2)/k^], where V_m_ represents the membrane potential, V_1/2_ represents the half-activation or half-inactivation potential, and k represents the slope factor. For the steady-state activation and inactivation curves, Y represents the relative conductance and relative current, respectively. The dose response curve was fitted using the Hill equation, (I_control_ − I_drug_)/I_control_ = A/[1 + (IC_50_/C_drug_)^n^], where I_control_ and I_drug_ represent the current in the absence and presence of drug, respectively; A represents the maximum inhibition rate; C_drug_ represents the drug concentration; and IC_50_ represents the half-maximal inhibitory concentration.

## Data Availability

All of the data in this study can be obtained from corresponding author upon reasonable request.
